# High interindividual variability in dose-dependent reduction in speed of movement after exposing *C. elegans* to shock waves

**DOI:** 10.3389/fnbeh.2015.00012

**Published:** 2015-02-06

**Authors:** Nicholas B. Angstman, Maren C. Kiessling, Hans-Georg Frank, Christoph Schmitz

**Affiliations:** Department of Neuroanatomy, Ludwig-Maximilians University of MunichMunich, Germany

**Keywords:** *C. elegans*, mild traumatic brain injury, blast trauma, shock wave, locomotion, paralysis, tracking

## Abstract

In blast-related mild traumatic brain injury (br-mTBI) little is known about the connections between initial trauma and expression of individual clinical symptoms. Partly due to limitations of current *in vitro* and *in vivo* models of br-mTBI, reliable prediction of individual short- and long-term symptoms based on known blast input has not yet been possible. Here we demonstrate a dose-dependent effect of shock wave exposure on *C. elegans* using shock waves that share physical characteristics with those hypothesized to induce br-mTBI in humans. Increased exposure to shock waves resulted in decreased mean speed of movement while increasing the proportion of worms rendered paralyzed. Recovery of these two behavioral symptoms was observed during increasing post-traumatic waiting periods. Although effects were observed on a population-wide basis, large interindividual variability was present between organisms exposed to the same highly controlled conditions. Reduction of cavitation by exposing worms to shock waves in polyvinyl alcohol resulted in reduced effect, implicating primary blast effects as damaging components in shock wave induced trauma. Growing worms on NGM agar plates led to the same general results in initial shock wave effect in a standard medium, namely dose-dependence and high interindividual variability, as raising worms in liquid cultures. Taken together, these data indicate that reliable prediction of individual clinical symptoms based on known blast input as well as drawing conclusions on blast input from individual clinical symptoms is not feasible in br-mTBI.

## Introduction

The increasing prevalence of blast-related mild traumatic brain injury (br-mTBI) has become a considerable health and economic issue (Hoge et al., 2008). Although exact figures are unknown due to difficulties in identifying cases of mTBI, it has been speculated that as many as one in six troops in the Iraq and Afghanistan military conflicts may be affected (Associated Press, 2007). Due to the variety of ways that the brain can be injured through blast exposure, damaging mechanisms can be defined by the injurious aspect of blast exposure. Damage caused by components of the blast wave itself, namely high positive pressure consequent to the first part of the blast wave as well as cavitation consequent to the tensile (second) part of the blast wave, is referred to as a primary blast effect (Nakagawa et al., 2011; Goeller et al., 2012; Rosenfeld et al., 2013). Secondary (penetrating objects), tertiary (bodily impact with other objects), and quaternary (e.g. crushing injuries from falling objects) blast effects are also defined (DePalma et al., 2005; Scott et al., 2006). While injuries resulting from secondary, tertiary, and quaternary blast effects may be more straightforward in terms of diagnosis and prognosis, injuries resulting from primary blast effects are more difficult. Specifically, accurate assessment of blast wave exposure is nearly impossible, as symptoms are generally not externally expressed. Furthermore, there are a wide range of clinical symptoms (such as headaches, loss of vision, fatigue, confusion, and memory loss) observed in br-mTBI that occur over both short- and the long-term scales (Heltemes et al., 2012). Much research has been done with the goal of furthering knowledge of the various aspects of the clinical picture and the neuropathology of br-mTBI (Cernak and Noble-Haeusslein, 2010; Morrison et al., 2011). There is still, however, little understanding of the mechanisms connecting initial trauma and expression of clinical br-mTBI symptoms, a limiting factor in the efficacy of br-mTBI protection and treatment.

High positive pressure followed by negative pressure that generates cavitation, as observed in blast waves, is also associated with therapeutic (extracorporeal) shock waves used in medicine for cracking kidney stones (Rassweiler et al., 2011) and treating tendinopathies, non-unions, and other injuries of the musculoskeletal system (Wang, 2012; Schmitz et al., 2013; Speed, 2014). It has also been demonstrated that the production of cavitation by shock waves can be highly reduced through propagation of shock waves through polyvinyl alcohol (PVA) rather than a control of frog Ringer's solution (Schelling et al., 1994). Specifically, PVA has high viscosity and very low cavitation activity (Hayakawa, 1991), and nearly the same acoustic impedance as water (Delius and Gambihler, 1992). Accordingly, PVA attenuates shock waves only very slightly more than water (Robinson and Kossoff, 1978). Hence, the exposure of *C. elegans* to shock waves in PVA enables the testing of the hypothesis that cavitation is an injurious component of primary blast waves.

Current *in vivo* models of br-mTBI generally investigate pathology in higher organisms (e.g., mice, rats, pigs) exposed to mock blasts, but generally are limited in sample size (Morrison et al., 2011). Typical *in vitro* methods of investigating br-mTBI use neuron cell cultures to investigate cellular and molecular cascades that are typically observed in mTBI, but the reproduction of such cascades is not done using known br-mTBI inducing causes (Morrison et al., 2011). *Caenorhabditis elegans* worms are widely used as a model organism alternative to vertebrate animals. Offering advantages including, but not limited to easy maintenance and handling, forward, and reverse genetic manipulability (Jorgensen and Mango, 2002), and a fully known nervous system connectome of 302 neurons (White et al., 1986), *C. elegans* worms are ideal for large population analysis of behavior. The simplicity of the *C. elegans* nervous system has the potential to link change in behavior to observed cellular change at a much more accessible level than in higher organisms. The possibility to investigate br-mTBI at a simple, high-throughput level makes *C. elegans* a useful model organism in the attempt to connect the bridge between cause and effect in br-mTBI.

In the present study we have developed a novel high-throughput method for br-mTBI research by exposing *C. elegans* worms to therapeutic shock waves in a manner that utilizes only primary blast effects to model mTBI. This novel method, by combining high throughput methods and analysis of *C. elegans* behavior, provides new, promising opportunities to study primary blast effects and their role in causing br-mTBI. We also demonstrate that cavitation contributes significantly to the damaging effect of shock waves on *C. elegans*. Here we tested the hypothesis that direct relationships exist between individual alterations of behavior of *C. elegans* and known blast input, which allows conclusions to be drawn on blast input from individual alterations of behavior.

## Materials and methods

### Nematodes

Wild type (N2, Bristol) *Caenorhabditis elegans (C. elegans)* and OP50 *Escherichia coli* were obtained from the Caenorhabditis Genetics Center (Minneapolis, MN). Liquid cultures of *C. elegans* fed with *E. coli* were maintained in 500 ml baffled flasks with 250 ml S-Medium (Sulston and Brenner, 1974) with 0.1% Tween 20. Cultures were kept at 24°C in an incubated shaker (NB-205V, N-Biotek, South Korea).

Cultures with many gravid hermaphrodites were selected, poured into a 500 ml separatory funnel (Nalgene, Rochester, NY), and allowed to settle for 15 min. The first 35 ml from the funnel, containing settled worms, were collected in a 50 ml tube. Eggs were then separated from stock liquid cultures using sodium hypochlorite treatment. Synchronized worms were obtained by allowing liquid cultures of eggs to hatch overnight and arrest at the L1 stage. Starved L1 larvae were fed using concentrated OP50 bacteria and allowed to grow at 24°C in S-medium with 0.1% Tween 20 to reach the young adult stage. After 46 h, young adult worms were extracted from culture using a separatory funnel, cleaned using the sucrose floatation method (Portman, 2006), and transferred to S-Medium.

In order to determine the influence of raising worms in liquid cultures on the results of shock wave exposure, we also raised worms on NGM agar plates. To this end, starved L1 larvae were pipetted from liquid cultures on to four NGM agar plates seeded with OP50 *E. coli* at the same time as the addition of *E. coli* to the synchronous liquid culture. Plates were then placed alongside the liquid culture in the incubated shaker at 24°C. In order to prepare for assaying, worms were washed off of plates with S-Medium with all following steps performed exactly the same as with the worms raised in liquid cultures.

### Application of shock waves

Synchronized, young adult worms of approximately 900–1000 μm in length were diluted to approximately 2 worms/μl S-Medium. In order to avoid contamination of adjacent probes, 10 μl of the worm stock were pipetted into non-adjacent U-bottom, 330 μl volume wells of a 96-well plate (VWR, Radnor, PA). Wells were then filled to 300 μl with S-Medium, or in the case of the cavitation assay, approximately 31,000 g/mol PVA (Mowiol 4-88, Karl Roth, Karlsruhe, Germany).

**Figure 1:**
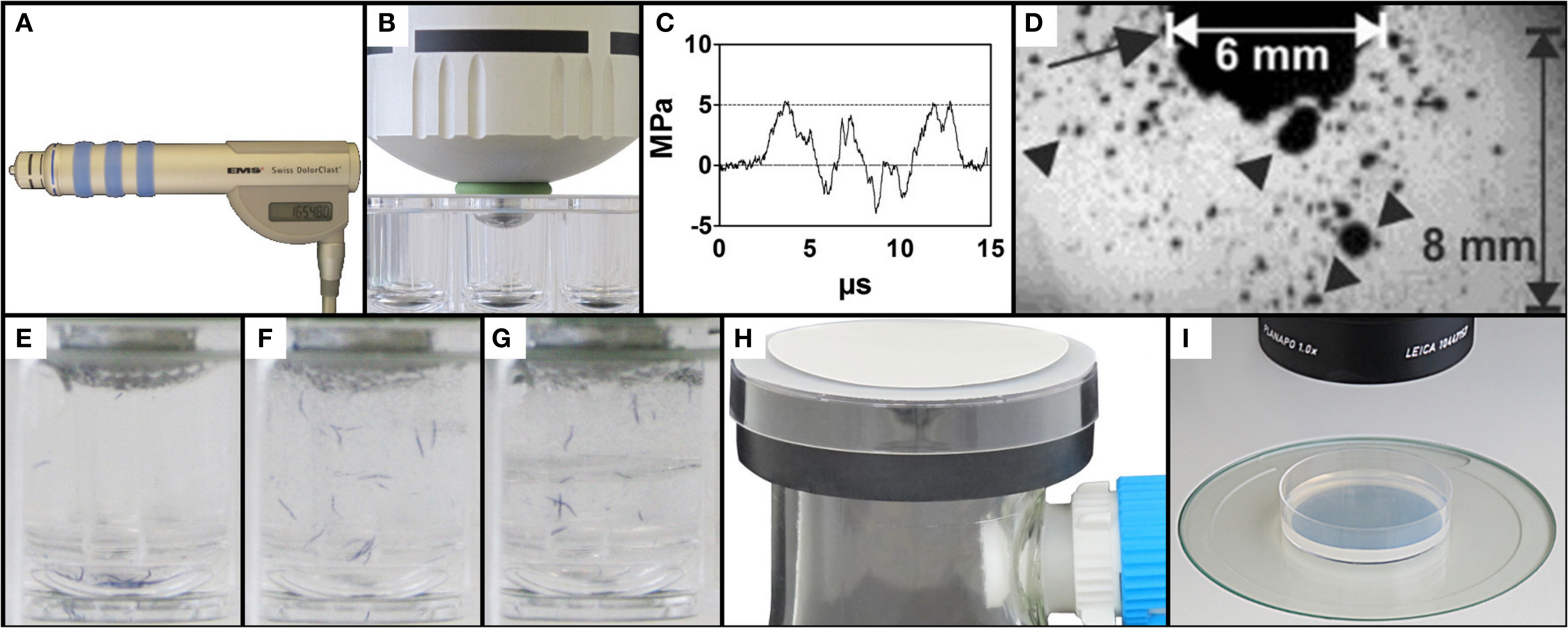
**Exposure of *C. elegans* to therapeutic shock waves**. **(A)** Handpiece of the Swiss DolorClast therapeutic shock wave device used in the present study. **(B)** Placement of the shock wave device 6-mm applicator tip and specialized O-ring in a single well of a 96-well plate. **(C)** Pressure as a function of time of the shock waves applied in the present study. Measurements were taken at 5 mm distance to the applicator with a fiber optic probe laser hydrophone (FOPH, 2000; RP Acoustics, Leutenbach, Germany) coupled to an oscilloscope (LeCroy 9361; LeCroy, Chestnut Ridge; NY). **(D)** Cavitation bubbles (arrowheads) produced by the shock waves applied in the present study. The arrow points to the 6-mm applicator of the shock wave device. The size of the cavitation field generated by the shock wave device as operated in the present study guaranteed that each worm was always exposed to cavitation when subjected to a shock wave, regardless of the position of the worm within the well. The image was taken with a high-speed CCD camera (Photron Ultima APX; Photron, Tokyo, Japan) with a framing rate of 300,000 frames per second and an exposure time of 1/2700,000 s. (**E**–**G**) Mixing property of shock wave application as shown by position of blue stained worms following 0, 10, and 50 shock waves, respectively, ensuring random, uniform exposure of *C. elegans* worms to shock waves. (**H**) Rapid-transfer method using membrane filters and vacuum suction to transfer *C. elegans* worms from liquid media to agar-plates in a matter of seconds. (**I**) Position of a worm-containing agar-plate under a dissecting microscope prepared for video capture.

The handpiece of a therapeutic (extracorporeal) shock wave device (Swiss DolorClast; Electro Medical Systems, S.A., Nyon, Switzerland; Figure 1A) was set vertically in a drill stand (Wolfcraft, Kempenich, Germany). The Swiss DolorClast generates therapeutic shock waves ballistically, i.e., by accelerating a projectile to strike an applicator, which transforms the kinetic energy of the projectile into a radially expanding pressure wave (Gerdesmeyer et al., 2008; Császár and Schmitz, 2013; Schmitz et al., 2013). The interior (6 mm internal diameter by 2 mm thick) rubber O-rings of the 6-mm applicator of the handpiece of the Swiss Dolorclast were replaced with identically sized 6 × 2 fluorinated rubber O-rings (Vi 975,G/FKM 75, C. Otto Gehrckens, Pinneberg, Germany) to better resist deterioration. A 5.5 × 2 fluorinated rubber O-ring (Vi 670/FKM 80, C. Otto Gehrckens) was placed externally around the applicator to provide a tight connection with the well plate, guarding against loss of sample. For dose-response analysis, the recovery assay, and the cavitation assay, the air pressure of the device was set to a constant 2 bar (yielding an energy flux density of the shock waves of 0.016 mJ/mm^2^ at a distance of 5 mm to the applicator). The application frequency was set to 5 Hz in all studies.

The shock wave applicator was slowly lowered into the center of a well and fixed in place once the downward pressure from the handpiece prevented lateral movement of the 96-well plate (Figure 1B). Proper placement of the device was confirmed by the direct connection of the applicator tip to the liquid medium, absent of air bubbles. Upon confirmation that the outer O-ring laid flush on top of the edge of the appropriate well, each sample of worms was exposed to a certain number of shock waves ranging from 0 to 500 impulses. The shock waves (Figures 1C,D) produced a mixing effect on the liquid sample, ensuring that all worms in the well were exposed to shock waves (Figures 1E–G and Video S1). Following application, the handpiece was slowly raised from the 96-well plate and the applicator was cleaned before further usage to prevent sample contamination. Worms were allowed to recover from shock wave application in the well for between 0 and 180 min. For dose-response analysis, 15 groups of worms were exposed to different shock wave doses (between 0 and 500 shock waves) and no recovery time was allowed. Males were systematically removed through manual video observation from the data set, occurring twice in the dose response analysis and not at all in the other assays. Data from 2285 worms were included in the dose-response experiment. A total of 30 different groups of worms were assayed in the recovery assay (six different numbers of shock waves applied times five different recovery periods), with data from a total of 3322 worms included in the experiment. In the cavitation assay, 727 worms were included in the experiment.

### Rapid transfer of worms from liquid to agar plates

In order to rapidly transfer *C. elegans* worms from the (three-dimensional) liquid medium to (quasi-two-dimensional) agar plates, a modified membrane filter-vacuum filtration system was created (Figure 1H; Video S2). A 250 ml side-arm flask was attached to a vacuum pump (BVC-21, Vacuubrand, Wertheim, Germany) and fitted with a rubber safety cuff (E350.1, Carl Roth) with grease on both sides to improve the vacuum seal. The top of a 6 cm plastic petri dish with an approximately 1 cm diameter hole in the middle was placed on top of the safety cup. Thin mesh was sealed over the hole to help prevent bubbling of the above membrane during vacuum filtration. A polyethersulfone Millipore Express PLUS Membrane (47 mm diameter, 0.22 μm pore size, Millipore, Billerica, MA) was placed on top of the assembly shiny side up.

To prevent worms from sticking to pipette tips, 30 μl of S-Medium with 10% Tween 20 were added to each well. The contents were then removed from wells using a pipette. Over 5–10 s under vacuum suction, the contents were dispensed drop by drop on to the membrane. Drops were dispensed so that the liquid did not spread beyond the 1 cm hole in the plastic petri dish. Once the liquid passed through the membrane, the vacuum pump was turned off and the membrane was removed with forceps. The membrane was then flipped over and laid flat on a 6 cm NGM agar plate seeded with OP50 *E. coli*. The transfer process was completed upon removal of the membrane from the agar plate. Video S2 shows the entire procedure.

### Locomotion data collection

After the transfer of worms, NGM agar plates were placed under a dissecting microscope (MZ75, Leica, Wetzlar, Germany; equipped with a 1.0× PlanApo objective) with an LCD light source set to a color temperature of 2800 K (KL 1500, Schott, Mainz, Germany) (Figure 1I). Using a 5.0 megapixel, mono digital camera (Grasshopper 2, Point Grey Research, Richmond, BC, Canada) and the video capture function of the software, WormLab (Version 2.0.1, MBF Bioscience, Williston, VT), plates were aligned so that all worms were within the field of view of the camera. One minute long videos were then captured at 15 frames per second (FPS) with a resolution of 1280 × 960 pixels.

Using a horizontal mm ruler and the measure function of WormLab, videos were determined to have a scale of 8.37 μm/pixel. Accordingly, the field-of-view of the camera was 10.7 by 8.0 mm. Using the settings defined in Table S1, videos were tracked from frame 1 to 900 (covering a one-minute period with 15 FPS) using WormLab (Video S3). Worm mid-point (x, y) position data was exported following the completion of tracking.

### Data processing and analysis

Transformation of raw mid-point position (x, y) data was performed using Microsoft Excel 2010 (Microsoft, Redmond, WA). Worm tracks were only included in the final data set when position values were present for greater than 75 of the first 150 frames. This eliminated the possibility of double counting worms while also allowing a reasonable time window (6 s) to demonstrate movement.

From each worm track that met the inclusion parameters, average speed was calculated from up to 1 min tracked. One speed value, without regard to direction, was calculated for each second as the difference in distance over fifteen frames. Single-second speed values of greater than 500 μm/second were regarded as single frame tracking errors and thrown out of the data set (less than 0.1% of data points). The average of these values was then used to represent the worm's average speed. When reporting average speed of population groups, all qualifying worms were represented equally regardless of number of frames tracked.

In order to determine whether worms were moving or not, each included worm's distance from first frame tracked was calculated for each following frame tracked. Worms that, at any point during tracking, reached a distance of greater than 100 μm from the first frame tracked were considered to be moving. Likewise, those that did not reach 100 μm from the first frame tracked were counted as paralyzed. As with speed, each qualifying worm was counted equally in the reporting of the relative number of worms rendered paralyzed, regardless of number of frames tracked.

### Statistical and dose-response analysis

Speed of movement in the dose-response assay was analyzed using the Kruskal–Wallis test with Dunns post test using GraphPad Prism version 5.04 for Windows (GraphPad Software, San Diego, CA). Distribution of speed within individual treatment groups for the dose-response assay was assessed using the Kolmogrov-Smirnov normality test using GraphPad Prism. The recovery, cavitation, and NGM agar plates vs. liquid cultures assays were assessed using univariate ANOVA using IBM SPSS Statistics (version 22, IBM Corp., Armonk, NY). Shock wave exposure and waiting period were fixed factors in the recovery assay, while shock wave exposure and medium of exposure were fixed factors in the cavitation assay. Shock wave exposure and medium of growth were fixed factors in the NGM agar plates vs. liquid cultures assay. Speed of movement was used as dependent variables in all three assays. *Post-hoc* tests for pairwise comparisons were performed with Bonferroni multiple comparison tests.

For each dose group, distribution of averaged speed data of single worms was analyzed using the software, R (R Development Core Team, 2008) by calculation of medians, quartiles, means, and outliers using the Tukey method (Tukey, 1977). The average speed data of single worms were also used to determine the dose-response relationship based on the speed data of all worms in the various dose groups with R using the package, “drc” (Ritz and Streibig, 2005) with the LL.4 function (maximal and minimal values of y not fixed). The fractions of moving and affected worms were calculated for each dose group using Microsoft Excel, and used as the input for binomial dose-response analysis. Binomial dose-response analysis was performed in R using the package, “drc” (Ritz and Streibig, 2005) with the LL.2 function (binomial dose-response analysis with maximal y value of 1 and minimal y value of 0).

A *p*-value of 0.05 was used as the criterion for statistical significance.

## Results

### Dose-dependent behavioral response of *C. elegans* to shock wave exposure

**Figure 2:**
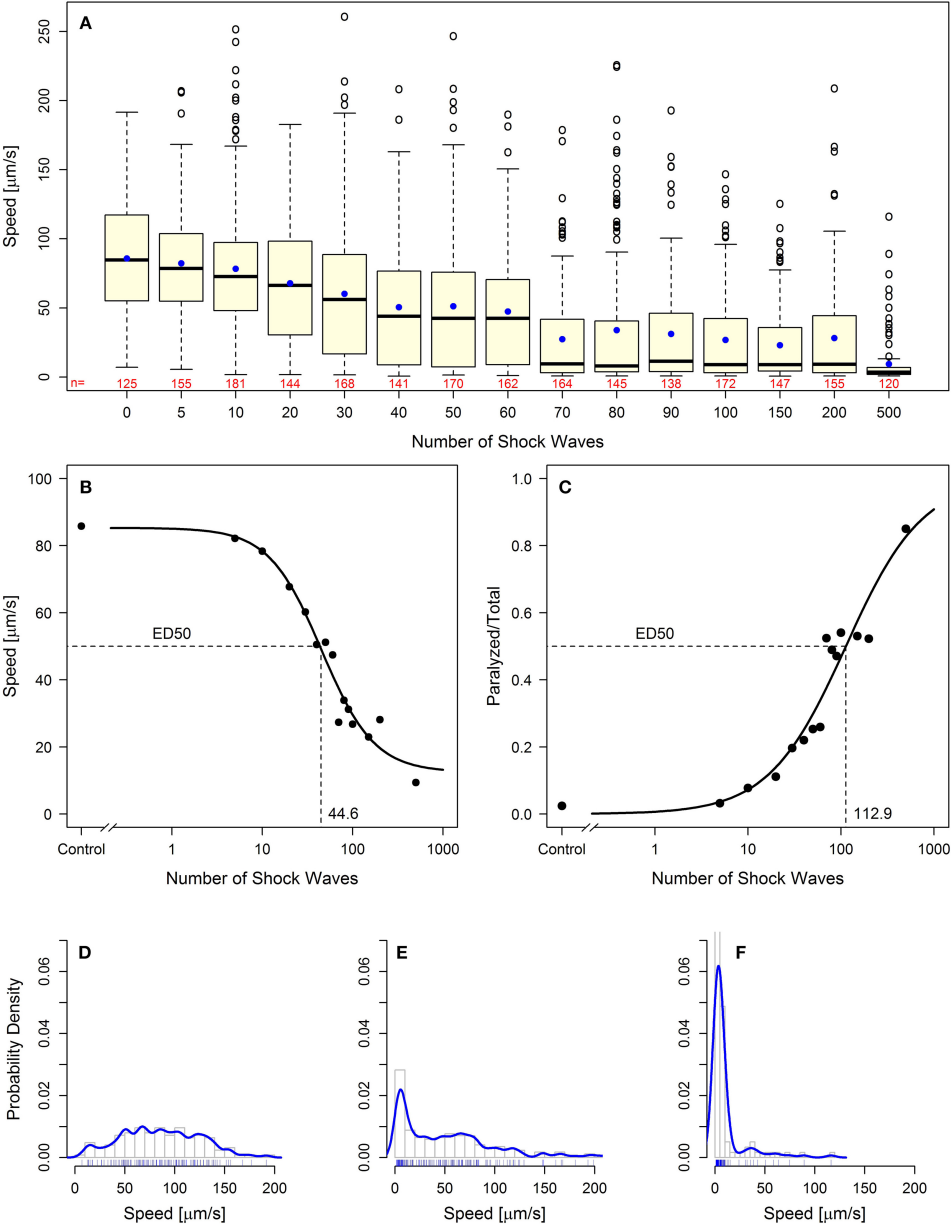
**Behavioral loss-of-function of *C. elegans* worms following shock wave exposure**. **(A)** Increased exposure to shock waves resulted in decreased mean speed of movement in all groups of worms exposed to at least 30 shock waves (*p* < 0.05). Represented as individual points, 97 positive-lying outliers were found within shock wave exposed groups of worms. Horizontal lines within the boxes represent median speeds, dots within the boxes average speeds, and box and whiskers were plotted using the Tukey method. The numbers below the boxes indicate the numbers of worms per group. **(B)** Worms exhibited dose-dependent response to shock wave application (ED_50_ = 44.6 shock waves) using the parameter “speed of movement” (calculated using the average speed of each individual worm). The dots represent average speed of the worms in each exposure groups. **(C)** Worms exhibited dose-dependent response to shock wave application (ED_50_ = 112.9 shock waves) using the parameter “relative number of worms paralyzed.” The dots represent the fraction of worms paralyzed in each exposure group. **(E)** Control worms showed a normal distribution of speed of movement. (**E**,**F**) Groups of worms exposed to more than five shock waves exhibited significant right-hand skew (*p* < 0.05). Probability density curves are shown for 50 **(E)** and 500 **(F)** shock waves, respectively.

Using two behavioral endpoints, we were able to demonstrate, for the first time, a dose-dependent effect of shock wave exposure on *C. elegans*. Increased exposure to shock waves resulted in statistically significantly (*p* < 0.05) decreased mean speed of movement while simultaneously increasing the proportion of worms rendered paralyzed (Figure 2A; Videos S4, S5; see also Figures 4A–C). Both parameters exhibited sigmoidal dose-response relationship (Figures 2B,C), and the half-maximal effective doses (ED_50_) at EFD = 0.016 mJ/mm^2^ (measured at a distance of 5 mm to the applicator) and shock wave frequency of 5 Hz were calculated to be 44.6 and 112.9 shock waves for speed of movement and the proportion of worms rendered paralyzed, respectively (Figures 2B,C).

Dose dependence of the shock wave effect was observed on a population-wide basis. Within each dose level there were large interindividual differences in speed of movement observed. Across 15 dose groups, a total of 97 worms were labeled as outliers using Tukey's method for outlier detection (Figure 2A) (Tukey, 1977). Further investigation of the distribution of worm speed of movement within dose groups showed that all groups of worms exposed to more than five shock waves exhibited statistically significant (*p* < 0.05) right handed skew (Kolmogorov–Smirnov test; Figures 2D–F). Accordingly, high interindividual variability within shock wave exposure groups was observed in *C. elegans* even though the subjects were genetically identical and exposed to shock waves under consistent, controlled conditions.

### *C. elegans* recover locomotive ability

**Figure 3:**
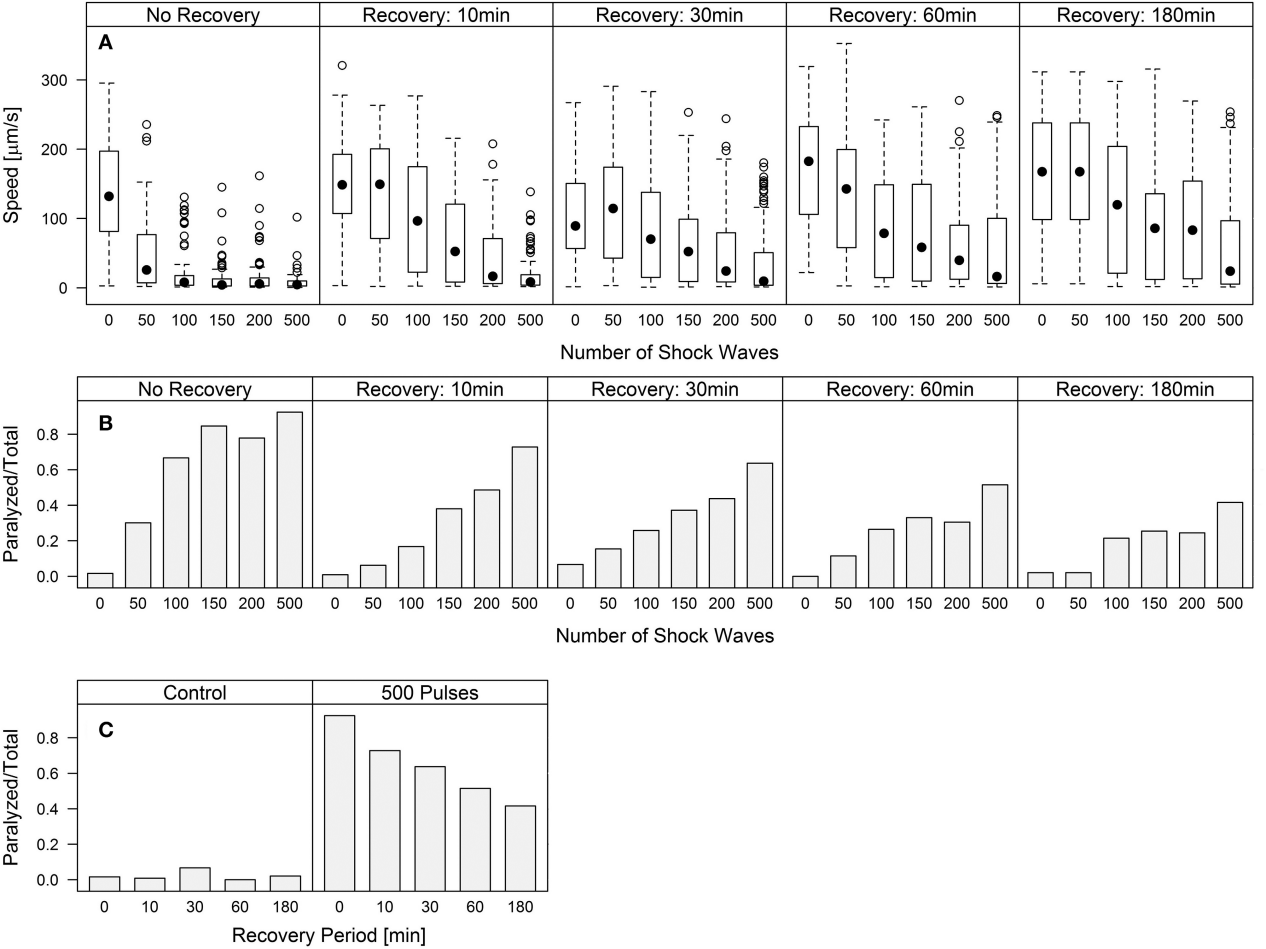
***C. elegans* regain behavioral function following the application of shock waves**. **(A)** Increased shock wave exposure reduced average worm speed (*p* < 0.001), while increased recovery period of up to 180 min resulted in increased speed (*p* < 0.001) **(B)** Fraction of worms paralyzed decreased over a 180 min recovery period. Regaining of function was observed in all groups after only 10 min, while control worms exhibited no noticeable difference between groups. **(C)** Worms exposed to 500 shock waves demonstrated gradually increasing recovery over four time points up to 180 min.

Worm speed of movement over increasing waiting periods after shock wave exposure approached control values, indicating a recovery of function (Figure 3A). Univariate analysis of variance (with shock wave exposure and waiting period as fixed factors, and speed of movement as dependent variable) showed that both the amount of shock waves and the waiting period had statistically significant (*p* < 0.001) influence on the speed of movement, as well as the combined shock wave/waiting period effect. Specifically, with increasing waiting period after shock wave exposure, worms showed, on average, higher speed of movement (Figure 3A). The proportion of worms rendered paralyzed showed the same statistically significant (*p* < 0.001) effects, with decreasing amounts of worms rendered paralyzed over a 180 min waiting period after shock wave exposure (Figure 3B). This was best exemplified by worms that were exposed to 500 shock waves. These worms showed marked improvement at each time point over the course of 3 h (Figure 3C, Video S6).

### Polyvinyl alcohol reduces shock wave effect on *C. elegans*

**Figure 4:**
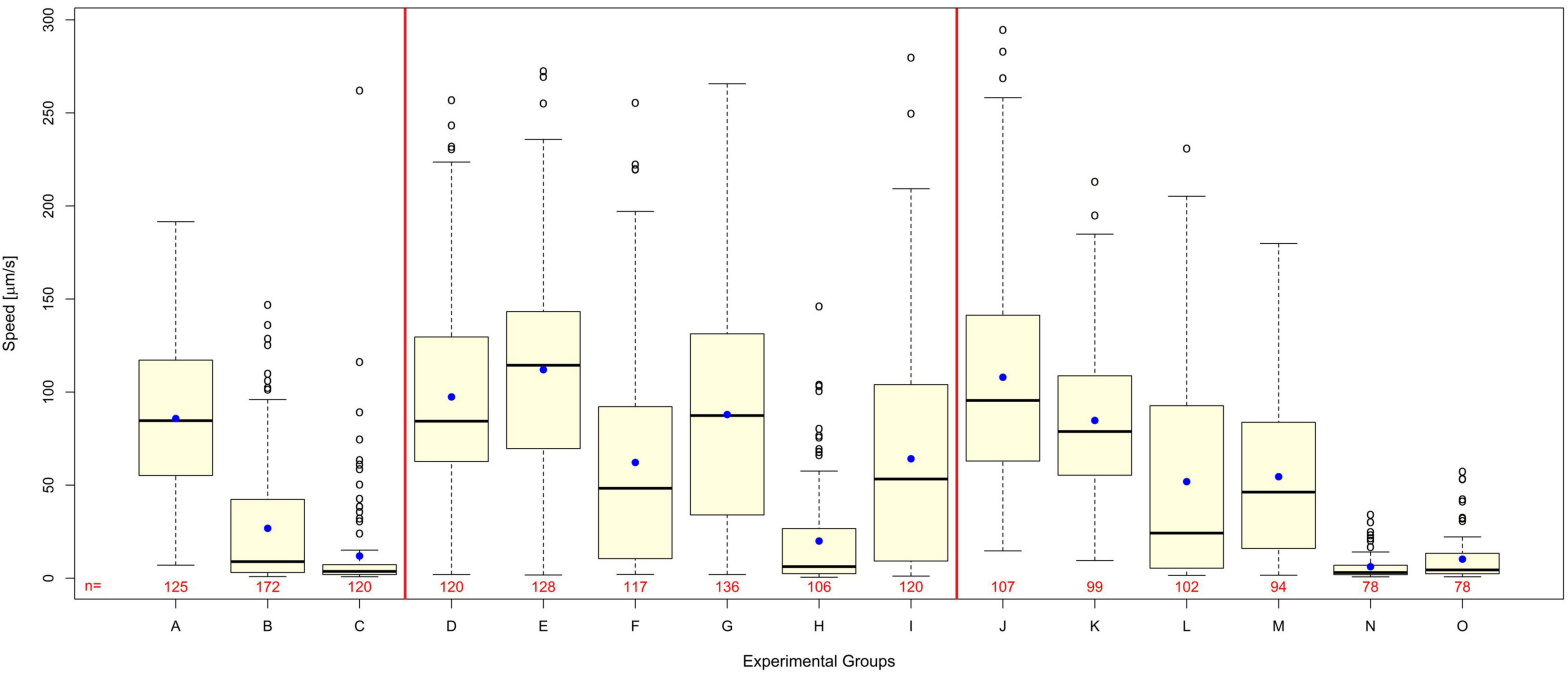
**Polyvinyl alcohol reduces shock wave effect on *C. elegans*, whereas shock wave exposed *C. elegans* grown on NGM agar plates exhibit similar behavioral change to shock wave exposed *C. elegans* raised in liquid cultures**. **(A–C)** Worms exposed to 0 **(A)**, 100 **(B)**, or 500 **(C)** shock waves in the main experiment of this study (these data are the same as those shown in Figure 2A and are displayed again here as a comparison only). **(D–I)** Worms exposed to 0 **(D)**, 100 **(F)**, or 500 **(H)** shock waves in S-Medium, compared to worms exposed to 0 **(E)**, 100 **(G)**, or 500 **(I)** shock waves in polyvinyl alcohol, a medium with low cavitation activity. Note that worms exposed to shock waves in polyvinyl alcohol demonstrated higher average worm speed than controls in S-medium (*p* < 0.001) immediately following application of shock wave. **(J–O)** Worms raised in liquid cultures and exposed to 0 **(J)**, 100 **(L)**, or 500 **(N)** shock waves in S-Medium, compared to worms grown on NGM agar plates and exposed to 0 **(K)**, 100 **(M)**, or 500 **(O)** shock waves. Note that shock wave exposure resulted in decreased speed of locomotion in both worms grown on NGM agar plates and worms raised in liquid cultures (*p* < 0.001). Medium of growth was not found to significantly affect speed of locomotion (*p* = 0.154), but the combined effect of medium of growth and shock wave exposure was found to be significant (*p* = 0.004). Horizontal lines within the boxes represent median speeds, dots within the boxes average speeds, and box and whiskers were plotted using the Tukey method. Positive-lying outlies are represented as individual points. It is of note that the high inter-individual variability reported in the main experiment (Figure 2) was neither specifically favored by one nature of medium in which worms were exposed to shock waves over another (S-Medium or polyvinyl alcohol) nor one culture media over another (S-medium vs. NGM agar plates). The numbers below the boxes indicate the numbers of worms per group.

**Figure 5:**
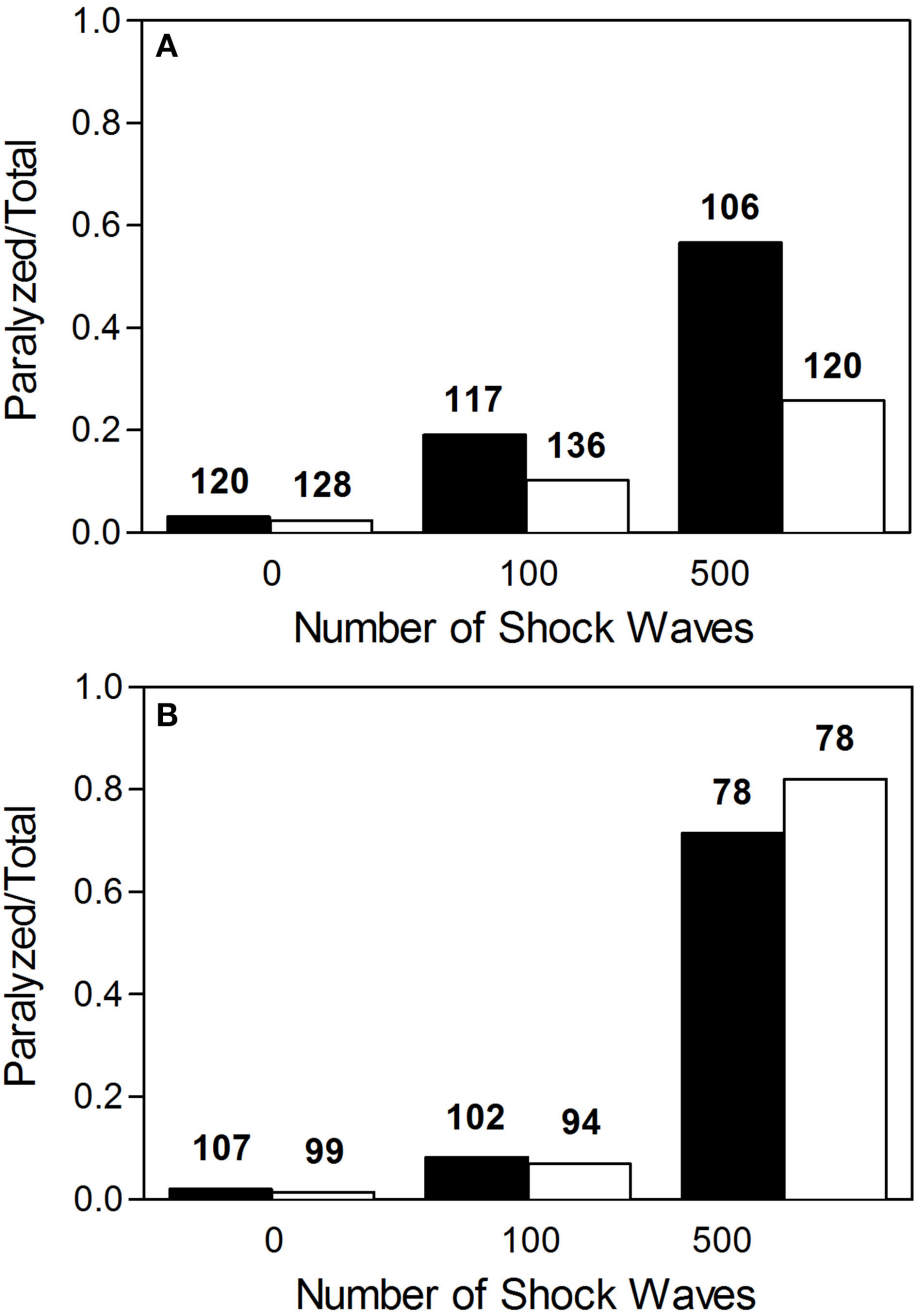
**Proportion of worms rendered paralyzed after exposure to shock waves. (A)** Fraction of worms paralyzed increased with added shock wave exposure, while the instance of paralysis was lower in worms exposed in polyvinyl alcohol (open bars) than in worms exposed in S-medium (closed bars). **(B)** Fraction of worms paralyzed increased with added shock wave exposure in both worms grown on NGM agar plates (open bars) and worms raised in liquid cultures (closed bars). The numbers above the boxes indicate the numbers of worms per group.

Worms exposed to shock waves in PVA showed increased speed of locomotion and decreased proportion of worms rendered paralyzed than controls in S-Medium (Figures 4D–I, 5A). Univariate analysis of variance (with shock wave exposure and medium of exposure as fixed factors, and speed of movement as dependent variable) showed that the amount of shock waves exposed, as well as the medium of exposure, had a statistically significant effect on speed of movement (*p* < 0.001). Furthermore, the combined shock wave and medium of exposure effect was statistically significant (*p* = 0.011). Post-hoc Bonferroni tests showed statistically significant differences between worms exposed to shock waves in PVA and worms exposed to shock waves in S-Medium after application of 100 (*p* < 0.001) and 500 shock waves (*p* < 0.01) but not after zero shock waves (*p* > 0.05). This indicates that, regardless of in which media, shock waves had a dose-dependent effect on worms. Control worms not exposed to shock waves indicated no ill-effects from PVA, as speed of locomotion was not found to be lower and proportion of worms rendered paralyzed was not higher. Worms exposed to 100 and 500 shock waves in PVA demonstrated higher speed of movement and lower proportion of worms rendered paralyzed than their respective S-Medium controls. Note that the high inter-individual variability reported in the main experiment (Figure 2) was not specifically favored by one medium in which worms were exposed to shock waves over another (S-Medium or polyvinyl alcohol).

### *C. elegans* grown on NGM agar plates exhibit similar behavioral response to shock wave

Worms grown on NGM agar plates showed response to shock wave similar to that of worms raised in liquid cultures (Figures 4J–O, 5B). Namely, univariate analysis of variance (with shock wave exposure and medium of growth as fixed factors, and speed of movement as dependent variable) showed a statistically significant decrease in speed of locomotion (*p* < 0.001) caused by exposure to shock wave. The speed of locomotion was not found to be significantly different between worms grown on NGM agar plates and worms raised in liquid cultures (*p* = 0.154), while the combined effect of shock wave and medium of growth was significant (*p* = 0.004). *Post-hoc* Bonferroni tests showed statistically significant differences between worms grown on NGM agar plates and worms raised in liquid cultures after application of zero (*p* < 0.001) but not after 100 shock waves (*p* > 0.05) or 500 shock waves (*p* > 0.05). Note that the high inter-individual variability reported in the main experiment (Figure 2) was not specifically favored by one nature of culture media over another (S-medium vs. NGM agar plates).

## Discussion

The results of the present study can be summarized as follows: *C. elegans* worms exposed to shock waves generated with a therapeutic shock wave device demonstrate, within a population group, (i) dose-dependent reduction in mean speed of movement and (ii) dose-dependent increase in percentage of worms rendered paralyzed. Both effects were reversible and represented a species-specific behavioral outcome that is comparable to that of higher organisms. Importantly, individual speed of movement showed high interindividual variability within shock wave exposure groups despite the fact that all exposed subjects were genetically identical and exposed to shock waves under consistent, highly controlled conditions. Accordingly, our null hypothesis was rejected. Furthermore, cavitation appears to be a significant injurious component of the shock waves used in the present study.

With regard to the validity of our novel C. *elegans*/shock wave model of br-mTBI, we aimed to produce symptoms in *C. elegans* that were measurable, like in humans, using a behavioral readout. In humans, mTBI is characterized by a wide range of short- and long-term neurologic symptoms such as headaches, loss of vision, fatigue, confusion, memory loss, and temporary loss of consciousness, among others (Heltemes et al., 2012). Using the behavioral endpoint of locomotion that is well-established in the literature (Rajini et al., 2008), we showed that *C. elegans* display reversible effects of paralysis following shock wave exposure. While much simpler than the complex behavioral outcomes observed in humans and mammalian models, the effects observed in *C. elegans* have—to some extent—similar characteristics as those observed in humans and mammalian models in that they contain a loss-of-function and a recovery. Moreover, the cause is analogous to hypothesized causes of br-mTBI. Specifically, the shock waves produced by the therapeutic shock wave generator used in the present study (i) have a duration on the order of microseconds, (ii) are characterized by a high positive pressure peak (approximately 5 MPa at a distance of 5 mm to the applicator in the present study) with (iii) an extremely short rising period (on the order of less than 1 μs), (iv) a high positive energy flux density (approximately 0.016 mJ/mm^2^ at a distance of 5 mm to the applicator in the present study), and (v) a final tensile period that causes cavitation (Figures 1C,D). These characteristics are shared with those of blast overpressure waves (also known as shock waves), a possible inducer of mTBI (Chavko et al., 2007; Elder and Cristian, 2009). Particularly, cavitation has been hypothesized to be a substantial factor in the causation of br-mTBI (Goeller et al., 2012). Thus, we have created a model in which behavioral effects with similar characteristics to those observed in humans and mammalian models of br-mTBI (loss-of-function, recovery) were observed in *C. elegans*. Furthermore, such effects were observed after exposure to shock waves that have similar physical characteristics to shock waves that are hypothesized to induce br-mTBI in humans.

Cernak and Noble-Haeusslein (2010) have proposed the following criteria for blast-related neurotrauma models: (i) the injurious component of the blast should be clearly identified and reproduced in a controlled, reproducible, and quantifiable manner; (ii) the inflicted injury should be reproducible, quantifiable, and mimic components of human blast-induced neurotrauma; (iii) the injury outcome established based on morphological, physiological, biochemical, and/or behavioral parameters should be related to the chosen injurious component of the blast; and (iv) the mechanical properties (intensity, complexity of blast signature, and/or its duration) of the injurious factor should predict the outcome severity. Our novel *C. elegans*/shock wave model of br-mTBI fully meets the first and third of these criteria. As our data showed that the inflicted injury was quantifiable but only reproducible on a statistical basis within a population group, the second and fourth of these criteria match our results on a population basis only. In accordance with this, predicting alterations in speed of movement of a single worm was neither possible based on the number of applied shock waves nor the recovery time. Nor was it possible to determine the number of applied shock waves and the recovery time based on the speed of movement of a single worm. These observations may have far-reaching implications for br-mTBI research, diagnosis, and therapy in humans. It thus becomes greatly important to determine what factors, outside of a known primary blast input and genetic heterogeneity (both are controlled in this model of br-mTBI), result in the considerable prognostic variability seen in human patients with br-mTBI (Heltemes et al., 2012).

In humans, rapid diagnosis of TBI severity is recommended in order for proper initial treatment and expectancy of forthcoming effects (Department of Defense and Department of Veterans Affairs, 2013). In order to do so, initial evaluation of mTBI severity is typically performed using behavioral endpoints. Specifically, the U.S. Department of Defense and Department of Veteran Affairs (Washington, DC, USA) has recommended assessing mTBI severity by combining the scores of three tests to classify mTBI as mild, moderate, or severe (Department of Defense and Department of Veterans Affairs, 2013). The model bases classification on duration of amnesia, reactions to stimuli (verbal, motor, and eye-opening), and duration of loss of consciousness. Thus, behavior is considered an extremely important parameter in determining mTBI severity in humans. The results of the present study indicate that mTBI severity can indeed best be predicted by clinical analysis and not—on an individual basis—by knowledge of the severity of the initial traumatizing impact. Although genetic variation has been proposed as a possible factor in mTBI pathology (Jordan, 2007; Waters et al., 2013), the discovery made in the present study furthers the importance of determining what factors beyond genetic difference contribute to the pathogenesis of br-mTBI in humans. Furthermore, it shows that efforts to base treatment regimens on the magnitude of exposure may be less fruitful than one might expect due to the lack of predictability of outcome. The findings in the present study are in line with clinical experience that br-mTBI remains best evaluated clinically on a patient-by-patient basis rather than based on known stimulus.

Both *in vitro* and *in vivo* methods have been developed to model mTBI; some were specifically developed to model br-mTBI. *In vitro* models of mTBI generally attempt to reproduce and investigate the cascades of molecular and cellular events that follow mTBI-induction (reviewed in Morrison et al., 2011); *in vivo* models, usually exposing mammals to blast or blunt trauma, mainly focus on the investigation of mTBI-related neuropathology (reviewed in Cernak and Noble-Haeusslein, 2010).

Mechanical stress applied to an *in vitro* isolation of neurons, although not produced using known mTBI inducers, allows for the observation and analysis of specific pathways that lead to observed mTBI effects. By limiting conditions and pathways, *in vitro* methods can be useful for the understanding of very specific mechanisms. However, these methods, while reproducible and potentially high-throughput, are incomplete models of mTBI. Molecular and cellular changes can be observed, but these changes are not directly linked to behavior. Our novel *C. elegans*/shock wave model of br-mTBI shares the same advantages of *in vitro* methods in terms of being high-throughput and reproducible, but also provides a dynamic model using an *in vivo* system with behavioral readout. Combined with the fact that *C. elegans* has a fully known nervous system, *C. elegans* can be used to investigate individual neurons and other cellular/tissue effects, while linking such changes on an individual basis to behavioral output (de Bono and Maricq, 2005). Furthermore, the ability of *C. elegans* to live in a liquid environment allows for easy, reliable, and reproducible application of both positive and negative pressure of shock waves. Thus, the novel *C. elegans*/shock wave model developed in the present study offers superior possibilities than established *in vitro* methods for br-mTBI research. It is of note that both worms grown on NGM agar plates and worms raised in liquid cultures showed the same general dose-dependent response to exposure of shock waves (Figures 4J–O, 5B). However, worms were not identical at baseline. Rather, worms raised in liquid cultures showed on average a higher speed of movement on agar plates than worms grown on NGM agar plates. The reason for this phenomenon is unknown and has to our knowledge not reported in the literature before. It was also this difference at baseline that caused the combined effect of shock wave and medium of growth to be statistically significant.

Mammalian models of br-mTBI have been established in multiple species. Mice and rats represent more than 90% of the corresponding literature, although other animals have also been used. In these models, mTBI-like conditions similar to those observed in humans have been successfully reproduced using various inducers. One of the most recent of these models was introduced by Goldstein et al. (2012). These authors developed a blast neurotrauma mouse model and found that head immobilization of the mice prevented blast-induced effects present following exposure without head immobilization. Thus, head movement as a result of blast wind was implicated as a damage-causing factor in the learning and memory deficits observed in specimen without head immobilization. Furthermore, pathology similar to that observed in br-mTBI victims was found in such mice. The findings by Goldstein et al. (2012) and others have furthered the understanding of mTBI neuropathology and have also led to the finding of biomarkers (Papa et al., 2012) and even possible pharmacological treatments that may reduce observed mTBI effects (Golding and Vink, 1994; Buki et al., 1999; Ohta et al., 2013). Although these findings represent progress, there remains a large void in the understanding of mTBI. The filling of such a knowledge gap may be slowed due to the difficulty of using mammalian models in a high-throughput manner. In mammalian models, subjects must be handled individually. Combined with the need that, in order to reliably determine ED_50_ doses, hundreds or even thousands of individuals must be investigated (as in the present study), mammalian models are simply inefficient. Even attempting to approach such a sample size is rendered impossible by concerns surrounding animal testing. To this end, our novel *C. elegans*/shock wave model of br-mTBI provides a viable alternative to conventional animal testing that reduces the need to subject mammals to pain, distress, and suffering. It should be noted that various methods have been proposed as alternatives to conventional animal testing, with the aim of replacing animals, reducing the numbers used, or refining the techniques to alleviate or minimize potential pain, distress and/or suffering (the so-called “3Rs” concept). Specifically, the U.S. National Toxicology Program, the U.S. Environmental Protection Agency, and other national and international agencies are committing significant resources toward the development of alternative species to be used as replacements for mammalian models in toxicological studies with particular emphasis on *C. elegans* (Boyd et al., 2010).

Our finding of outliers as an important endpoint of experimental br-mTBI research also required the analysis of thousands of *C. elegans* worms. Human outliers are known to exist (Heltemes et al., 2012) but–to our knowledge–have never been systematically addressed in br-mTBI research because of low sample sizes. On top of procedural and ethical issues, the increased sample-size ability of our novel *C. elegans*/shock wave model of br-mTBI provides the opportunity for in-depth investigation of subjects that display an unexpectedly positive or negative outlook. With sample sizes on the order of thousands combined with the presence of positive and negative outliers, this model provides a unique opportunity to reliably find an adequate sample of outliers. Side-by-side comparison of these outliers to worms that exhibit roughly the median effect could provide useful novel insight into the pathogenesis of br-mTBI.

The reduction in damaging effect on *C. elegans* in PVA indicates that cavitation is an important damage-causing component underlying the decrease of *C. elegans* locomotive ability. Cavitation has previously been shown to have an effect on nervous tissue, as Schelling et al. (1994) demonstrated reduced excitability in frog sciatic nerves following shock wave exposure in PVA as compared to control. Furthermore, cavitation is hypothesized to play an important role in the causation of br-mTBI (Goeller et al., 2012). This finding in *C. elegans* offers the potential to specifically investigate the effect of cavitation in an *in vivo* system. It is important to realize that the aforementioned recent br-mTBI model of Goldstein et al. (2012) does not offer this potential because it is technically nearly impossible to expose mice to shock waves in liquid media such as PVA that reduce cavitation. Hence, comparing molecular and cellular consequences of exposing *C. elegans* to shock waves (present study) with molecular and cellular consequences of exposing mice to blast events as done by Goldstein et al. (2012) may be completely misleading due to the fact that in the latter model, primarily tertiary effects (in this case head movement caused by blast wind) are to blame for observed phenomena. In the present study, however, primary blast effects (blast overpressure and cavitation) are implicated as the main damage causing factors.

In summary, exposing *C. elegans* worms to shock waves generated by a therapeutic shock wave device shares (i) similar physical characteristics of the applied shock waves to shock waves that are hypothesized to induce br-mTBI in humans, and (ii) species-specific behavioral characteristics similar to those observed in humans and mammalian models of br-mTBI, with known ED_50_ doses for two behavioral endpoints (average speed of movement; relative number of worms rendered paralyzed). Based on the observation of large interindividual variability (i.e. lack of predictable outcome based on known input), we hypothesize that br-mTBI treatment methodology based on measured blast input is an unreliable solution. This finding supports the clinical experience that br-mTBI patients are best treated on a patient-by-patient basis.

## Author contributions

Nicholas B. Angstman, Hans-Georg Frank and Christoph Schmitz conceived and designed the experiments, Nicholas B. Angstman performed experiments, and Nicholas B. Angstman, Maren C. Kiessling, Hans-Georg Frank and Christoph Schmitz analyzed data and wrote the manuscript.

## Funding

This work was supported by a grant from the Friedrich-Baur Foundation at the Ludwig-Maximilians University of Munich (to Maren C. Kiessling and Christoph Schmitz).

### Conflict of interest statement

Christoph Schmitz serves as paid consultant for Electro Medical Systems (Nyon, Switzerland), the manufacturer of the Swiss DolorClast that was used in the present study to generate shock waves, as well as paid consultant for MBF Bioscience (Williston, VT, USA), the manufacturer of the WormLab software that was used in the present study to analyze data. However, Christoph Schmitz has not received financial support directly or indirectly related to this manuscript. The authors declare that the research was conducted in the absence of any commercial or financial relationships that could be construed as a potential conflict of interest.

## Supplementary material

Video S1 **Uniform exposure of *C. elegans* to therapeutic shock waves**. Blue dyed worms demonstrate the mixing property of the therapeutic shock wave device.

Video S2 **Novel method for rapid transfer of *C. elegans* from liquid media to agar plates**. Transfer of worms took place in under 30 s—from time of well-extraction to placement of worms on ready-for-analysis agar plates.

Video S3 **Tracking of *C. elegans* movement using the WormLab software**. Video demonstrates tracking of control worms. Each worm assigned a number, green dot indicating mid-point of the worm.

Video S4 **Control sample of *C. elegans***. Worms exposed to zero shock waves.

Video S5 ***C. elegans* exposed to shock waves**. Worms not allowed recovery period following exposure to 500 shock waves.

Video S6 ***C. elegans* following post-shock wave recovery period**. Worms exposed to 500 shock waves and allowed to recover for 180 min before analysis.

Table S1 **Tracking Settings used in WormLab software**.
