# Supplementary material for: High interindividual variability in dose-dependent reduction in speed of movement after exposing C. elegans to shock waves
Source: Front Behav Neurosci. 2015 Feb 6;9:12. doi: 10.3389/fnbeh.2015.00012 (PMC4319468; doi:10.3389/fnbeh.2015.00012)
Supplement: Table S1 — Tracking Settings used in WormLab software. [file Table1.DOCX]

**Table S1 | Tracking Settings used in WormLab software (MBF Bioscience).**

| **Category** | **Setting** | **Value** |
| --- | --- | --- |
| Sequence | Captured Frame Rate | 15 FPS |
| Info | Scale | 8.37 µm/pixel |
| Image | Threshold Level^a^ | *Per Video* |
| Parameters | Image Mode | Dark Worms on a Light Background |
|  | Gradient Correction | Off |
|  | Smoothing (Gaussian Filtering) | Off |
|  | Fill Holes | Off |
| Detection | Detect Worms at the Edge of the Image | Off |
| Parameters | Area^b^ | *Per Video* |
|  | Length^b^ | *Per Video* |
|  | Width^b^ | *Per Video* |
|  | Width/Length Ratio^b^ | *Per Video* |
|  | Detection Frequency | 1 |
|  | Length Fitting | Off |
|  | Width Fitting | On |
|  | Use Whole Plate Mode | Off |
|  | Fitting Iterations | 80 |
|  | Spinal Axis Sample | 59 |
|  | Enforce Width Uniformity | Off |
| Tracking | Start Frame | 1 |
| Parameters | End Frame | 900 |
|  | Use Back Tracking | On |
|  | Track Worms at the Edge of the Image | Off |
|  | Max Tracked Hypotheses | 1 |
|  | Frames Worms Can Touch Boundary | 5 |
|  | Frames Worms Can Overlap | 50 |
|  | Position Tolerance | 0.30 |
|  | Shape Tolerance | 0.25 |
|  | Minimum Track Duration | Off |

^a^ Chosen to optimize representation of worms while limiting appearance of background

^b^ Automatically calculated based on manual identification of multiple worms
